# Diffuse alveolar hemorrhage after hematopoietic cell transplantation- response to treatments and risk factors for mortality

**DOI:** 10.3389/fonc.2023.1232621

**Published:** 2023-07-20

**Authors:** Michelle L. Schoettler, Christopher E. Dandoy, Anora Harris, Marilynn Chan, Keiko M. Tarquinio, Sonata Jodele, Muna Qayed, Benjamin Watkins, Pradip Kamat, Toni Petrillo, Jeremy Obordo, Christine S. Higham, Christopher C. Dvorak, Adrianna Westbrook, Matt S. Zinter, Kirsten M. Williams

**Affiliations:** ^1^ Division of Blood and Marrow Transplantation, Children’s Healthcare of Atlanta, Aflac Blood and Cancer Disorders Center, Emory University, Atlanta, GA, United States; ^2^ Cincinnati Children’s Medical Center, Division of Bone Marrow Transplantation and Immune Deficiency, University of Cincinnati School of Medicine, Cincinnati, OH, United States; ^3^ Pediatric Pulmonary Medicine, University of California, San Francisco, San Francisco, CA, United States; ^4^ Division of Critical Care Medicine, Department of Pediatrics, Children’s Healthcare of Atlanta, Emory University, Atlanta, GA, United States; ^5^ Pediatric Allergy, Immunology, and Bone Marrow Transplant Division, University of California, San Francisco, San Francisco, CA, United States; ^6^ Department of Pediatrics, Pediatric Biostatistics Core, Emory University, Atlanta, GA, United States; ^7^ Pediatric Critical Care, University of California, San Francisco, San Francisco, CA, United States

**Keywords:** diffuse alveolar hemorrhage (DAH), steroids, inhaled tranexamic acid (INH TXA), inhaled recombinant activated factor VIIa (INH fVIIa), transplant-associated thrombotic microangiopathy (TA-TMA), sinusoidal obstructive syndrome (SOS), non-relapse related mortality

## Abstract

Diffuse alveolar hemorrhage (DAH) is a life-threatening complication of hematopoietic cellular therapy (HCT). This study aimed to evaluate the effect of DAH treatments on outcomes using data from consecutive HCT patients clinically diagnosed with DAH from 3 institutions between January 2018-August 2022. Endpoints included sustained complete response (sCR) defined as bleeding cessation without recurrent bleeding, and non-relapse mortality (NRM). Forty children developed DAH at a median of 56.5 days post-HCT (range 1-760). Thirty-five (88%) had at least one concurrent endothelial disorder, including transplant-associated thrombotic microangiopathy (n=30), sinusoidal obstructive syndrome (n=19), or acute graft versus host disease (n=10). Fifty percent had a concurrent pulmonary infection at the time of DAH. Common treatments included steroids (n=17, 25% sCR), inhaled tranexamic acid (INH TXA,n=26, 48% sCR), and inhaled recombinant activated factor VII (INH fVIIa, n=10, 73% sCR). NRM was 56% 100 days after first pulmonary bleed and 70% at 1 year. Steroid treatment was associated with increased risk of NRM (HR 2.25 95% CI 1.07-4.71, p=0.03), while treatment with INH TXA (HR 0.43, 95% CI 0.19- 0.96, p=0.04) and INH fVIIa (HR 0.22, 95% CI 0.07-0.62, p=0.005) were associated with decreased risk of NRM. Prospective studies are warranted to validate these findings.

## Highlights

1. In 40 children with DAH after HCT, steroid treatment was associated with an increased risk of NRM (HR 2.25 95% CI 1.07-4.71, p=0.03).
2. Treatment with INH TXA (HR 0.43, 95% CI 0.19- 0.96) and INH fVIIa (HR 0.22, 95% CI 0.07-0.62) was associated with a lower risk of NRM.

## Background

Diffuse alveolar hemorrhage (DAH) is a rare complication of hematopoietic cell transplantation (HCT) associated with with high mortality (1–3). The pathophysiology of DAH is poorly understood but hypothesized to involve injury to the pulmonary endothelium from preparative agents, inflammation, and cytokine release (4, 5). There are no standard therapies for DAH (6). Historically, treatment included high-dose corticosteroids (3, 7, 8), though recent reports suggest this approach is associated with poorer survival after HCT (9–11).

Red blood cells are prone to hemolyze in patients with lung injury, and free heme released from these cells is highly reactive, contributing to additional lung damage (12–14). Thus, in addition to targeting drivers of DAH, cessation of bleeding may be an important component of treatment. Emerging evidence supports inhaled approaches to treat DAH, which minimize the risk of systemic thrombosis. Inhaled (INH) tranexamic acid (TXA) prohibits the conversion of plasminogen to plasmin, inhibiting fibrinolysis, and stabilizing clots and has shown excellent cessation of DAH (15), including in small cohorts of pediatric HCT recipients (2, 16). Recombinant activated factor VIIa (fVIIa) promotes hemostasis *via* tissue factor-dependent and independent pathways. Intrapulmonary administration of fVIIa has also halted pulmonary bleeding (17–20). While these studies demonstrate bleeding cessation, they have not shown an impact on survival in the HCT setting. This study aimed to evaluate the effect of DAH treatments on outcomes in a contemporary pediatric HCT cohort.

## Methods

In this IRB-approved retrospective study data were extracted from consecutive HCT patients clinically diagnosed with DAH between January 2018-August 2022 from 3 institutions, Children’s Healthcare of Atlanta, Cincinnati Children’s Medical Center, and the University of California, San Francisco. A sustained complete response (sCR) to treatment was defined as bleeding cessation without recurrent bleeding, a CR as bleeding cessation for ≥24 hours but with a subsequent recurrent bleed, and no response (NR) was continued bleeding or death with active bleeding. Acute graft versus host disease (aGVHD) was staged and graded using Glucksberg criteria. Systemic and pulmonary infections were identified by culture, PCR, or next-generation sequencing. Descriptive statistics were used to compare groups. Sub-distribution hazard models were used to generate hazard ratios (HR) for non-relapse mortality (NRM), treating relapse as a competing risk. SAS 9.4 (Cary, NC) was used, and statistical significance was set at 0.05.

## Results/discussion

Forty children developed DAH a median of 56.6 days post HCT (range 1-760). Each patient experienced 1-4 separate pulmonary bleeds with 27 (68%) incurring only one bleed. The first pulmonary bleed was diagnosed by bronchoscopy (n=24), blood in the endotracheal tube (n=14), hemoptysis (n=1), and lung tissue (n=1). The majority 35/40 of patients underwent allogeneic HCT; all 5 autologous recipients developed DAH post-second tandem HCT for neuroblastoma. Eighty- eight percent of patients (35/40) had at least one concurrent endothelial disorder, including transplant-associated thrombotic microangiopathy (n=30, 75%), sinusoidal obstructive syndrome (n=19, 48%) and acute graft versus host disease (n=10, 29%). Sixty percent (21/35) of patients had more than one endothelial disorder (**Supplemental Figure 1**). Twenty-three (58%) had a systemic infection within four weeks of DAH, and 20 (50%) had documented pulmonary infection at the time of bleed (**Table 1**).

**Table 1:** Characteristics of children with DAH post HCT by NRM versus Alive or Relapsed.

Variable	Alive/Relapsed (n=13, 34%), N (%)	NRM (n=27, 68%), N (%)	p-value
Age years (median, range)^+^	**3 (0-24)**	**11 (0.28- 20.5)**	**0.02**
Sex^^^ Female Male	10 (40) 3 (20)	15 (60) 12 (80)	0.29
Race^^^ White Black/African American Asian Other/Declined	**4 (17)** **4 (40)** **2 (67)** **3 (75)**	**19 (83)** **6 (60)** **1 (33)** **1 (25)**	**0.02**
Ethnicity^^^ Hispanic Non-Hispanic	2 (33) 11 (32)	4 (67) 23 (68)	1.0
HCT Indication^^^ Heme Malignancy Immune Def/Dys Non-malignant Heme Solid Tumor Neuro/Metabolic	6 (32) 1 (13) 1 (20) 4 (80) 1 (33)	13 (68) 7 (88) 4 (80) 1 (20) 2 (67)	0.16
HCT Type^^^ Allogeneic Autologous	**9 (28)** **4 (80)**	**26 (74)** **1 (20)**	**0.03**
Cell Source*^^^ Bone Marrow Peripheral Blood Umbilical Cord	**3 (14)** **3 (30)** **3 (75)**	**18 (86)** **7 (70)** **1 (25)**	**0.02**
Donor*^^^ Related Unrelated	3 (21) 6 (29)	11 (79) 15 (71)	0.71
HLAMismatch*^@^^ 8/8 7/8 ≤6/8	3 (15) 1 (17) 2 (40)	17 (85) 5 (83) 3 (60)	0.51
Preparative Intensity^^^ Myeloablative RIC Non-myeloablative	**12 (44)** **1 (8)** **0 (0)**	**15 (56)** **11 (92)** **1 (100)**	**0.04**
Acute GVHD Prophylaxis*^ CNI Methotrexate MMF Abatacept T-cell depletion	6 (21) 3 (20) 3 (21) 1 (33) 2 (40)	23 (79) 12 (80) 11 (79) 2 (67) 3 (60)	0.88
TA-TMA^^^	9 (30)	21 (70)	0.70
Eculizumab^^^	9 (35)	17 (65)	1.0
SOS^^^	4 (21)	15 (79)	0.19
Defibrotide^^^	4 (22)	14 (78)	0.31
Maximum aGVHD*^^^ Grade 0-2 Grade 3-4	7 (28) 2 (20)	18 (72) 8 (80)	0.69
Concurrent Systemic Infection^#%^^	8 (35)	15 (65)	1.0
Concurrent Pulmonary Infection^#%^^	7 (35)	13 (65)	1.0
Day first pulmonary bleed (median, range)	59 (52)	36 (163)	0.74
Number of pulmonary bleeds (median, range)^+^	1 (1-4)	1 (1-4)	0.53

*Allogeneic HCT recipients only (n=35), ^#^within 4 weeks of first pulmonary bleed, ^%^diagnosed via bacterial, viral, or fungal culture or next generation sequencing of BAL fluid. ^@^excluding cord blood, ^Fishers Exact Test, ^+^Wilcoxon-Rank Sum Test, Abbreviations: human leukocyte antigen (HLA), reduced intensity conditioning (RIC), graft versus host disease (GVHD), calcineurin inhibitor (CNI), mycophenolate mofetil (MMF), transplant associated thrombotic microangiopathy (TA-TMA), sinusoidal obstructive syndrome (SOS), graft versus host disease (GVHD), hematologic malignancy (heme malignancy), immune deficiency/dysregulation (immune def/dys), non-malignant blood disorder (non-malignant heme). Bold indicates significant differences.

**Figure 1:**
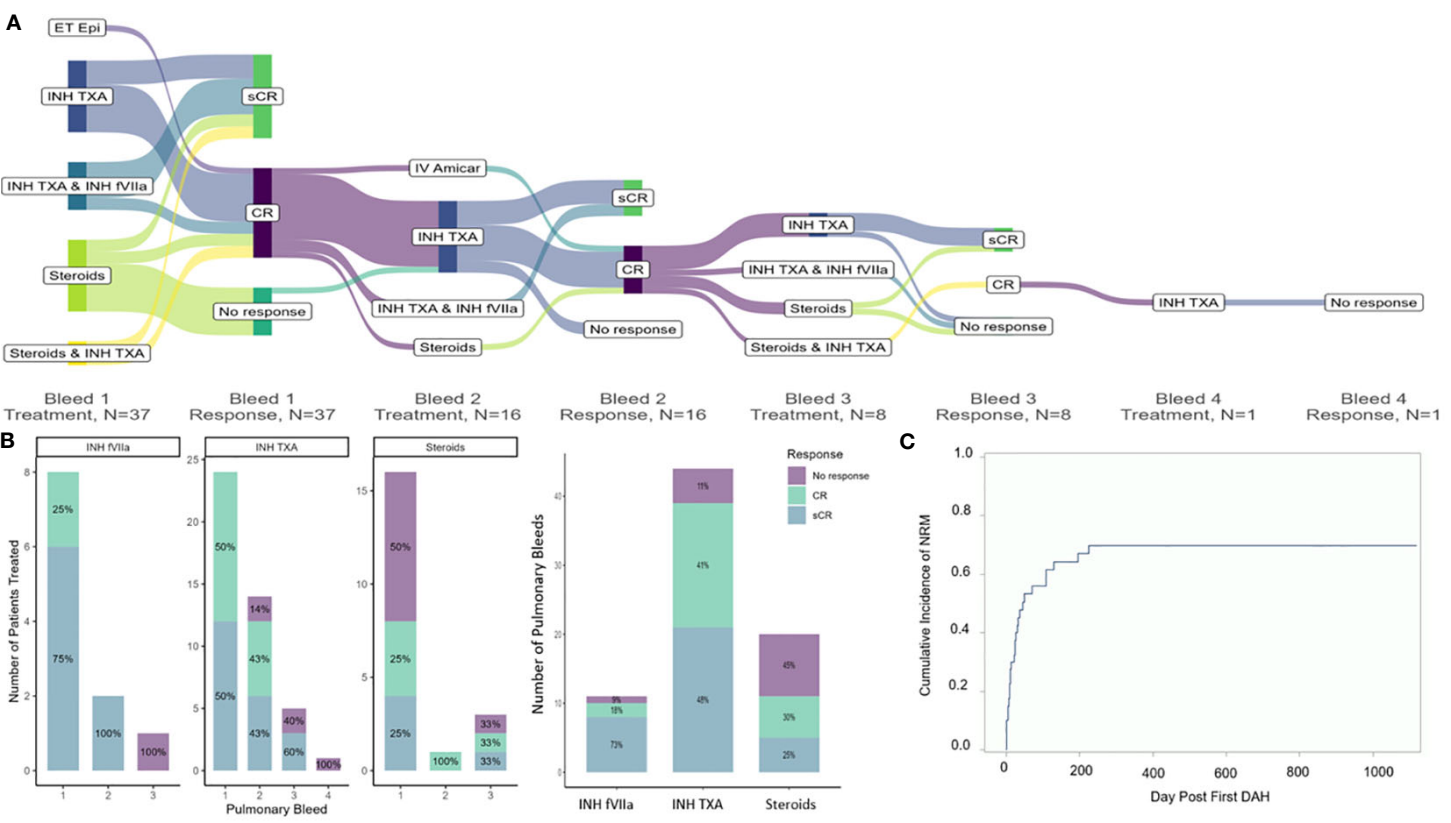
Treatment approaches **(A)**, response of pulmonary bleeds **(B)** and non-relapse related mortality **(C)**. **(A)** In this sankey diagram, combinations of treatments for DAH and the response of treatments are indicated for each pulmonary bleed. Not all bleeds were treated; 37 patients received treatment for first pulmonary bleed. Sustained complete response (sCR) was defined as cessation of bleeding without a rebleed. CR as cessation of bleeding for ≥24 hours, but with a recurrent bleed, and no response (NR) as continued bleeding or death with bleeding. **(B)** Response rates of each pulmonary bleed to each agent; notably, many patients received multiple agents. **(C)** Overall response to each agent. As above, multiple drugs were given concurrently; assessment of response was the same for all concurrently administered drugs.

**Figure 2:**
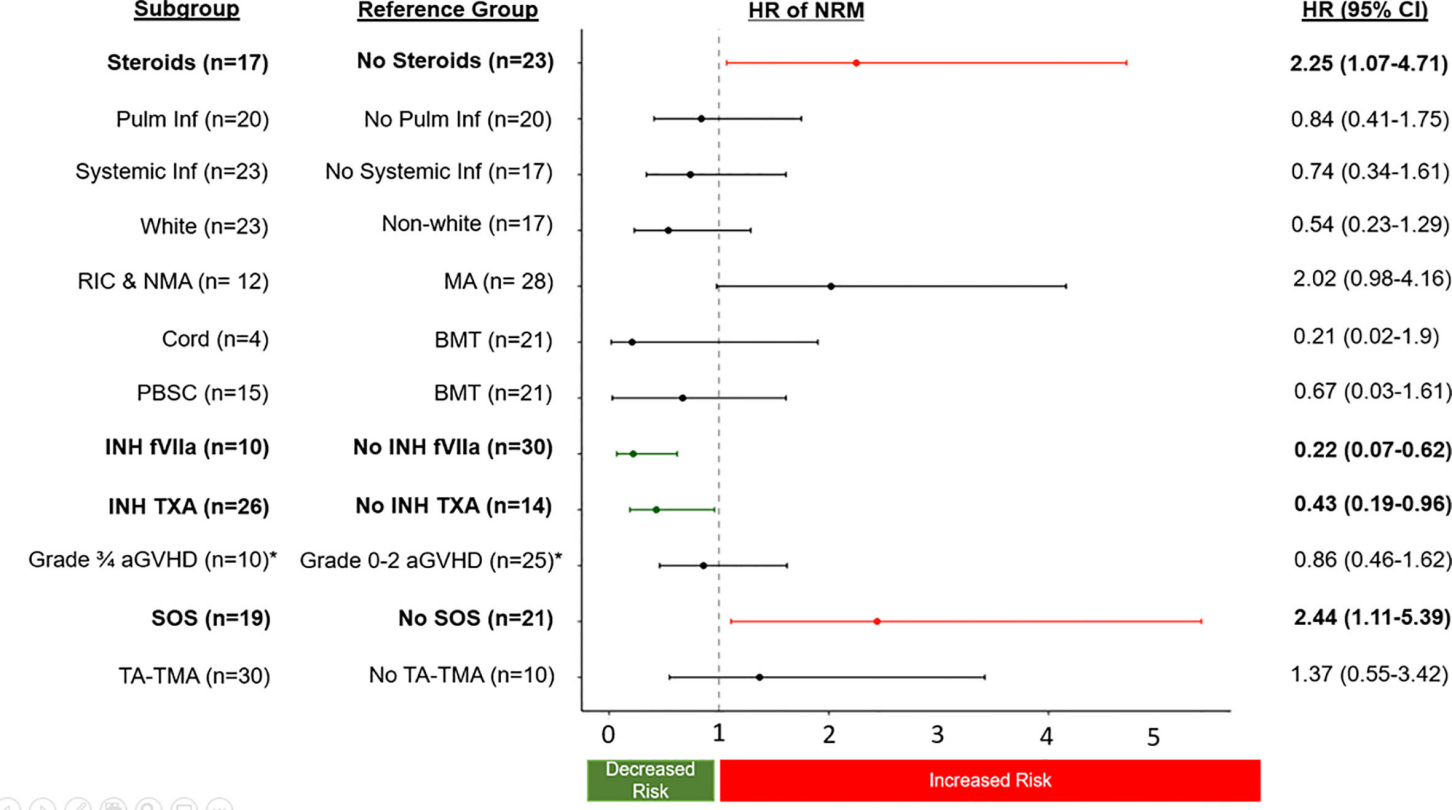
The sub-distribution HR of NRM (relapse competing risk) of transplant complications and treatment approaches for DAH. HR greater than 1 are associated with an increased risk of NRM, and less than 1 associated with a decreased risk of NRM. *only allogeneic patients were at risk and used in the analysis (n=35).

There were 60 separate pulmonary bleeds. Most patients received multiple treatments for each bleed (**Figure 1A**). Patients were most commonly treated with steroids (n=17), INH TXA (n=26), and INH fVIIa (n=10). While response rates varied, steroids had an overall sCR/CR of 55%, INH TXA 89%, and INH fVIIa 92% (p= 0.002, **Figure 1B**). NRM was 56 ± 8% and 70 ± 7% at 100 days and 1-year post first pulmonary bleed, respectively (**Figure 1C**). TA-TMA and grade III-IV GVHD were not associated with NRM. However, SOS (HR 2.44 95% CI 1.11-5.39, p=0.03) and steroid treatment (HR 2.25 95% CI 1.07-4.71, p=0.03) were associated with an increased risk of NRM. Treatment with INH TXA (HR 0.43, 95% CI 0.19- 0.96, p=0.04) and INH fVIIa (HR 0.22, 95% CI 0.07-0.62, p=0.005) were associated with decreased NRM (**Figure 2**). After adjusting for SOS, the only other variable significantly associated with NRM, the HR of NRM in those treated with steroids remained significantly higher (HR 2.35, 95% CI 1.14-4.88). To determine if infection impacted NRM risk in those treated with steroids, we adjusted for an identified systemic or pulmonary infection; the HR of NRM in children remained significantly higher in those treated with steroids (HR 2.2, 95% CI 1.0-4.81, p=0.05).

**Figure 3:**
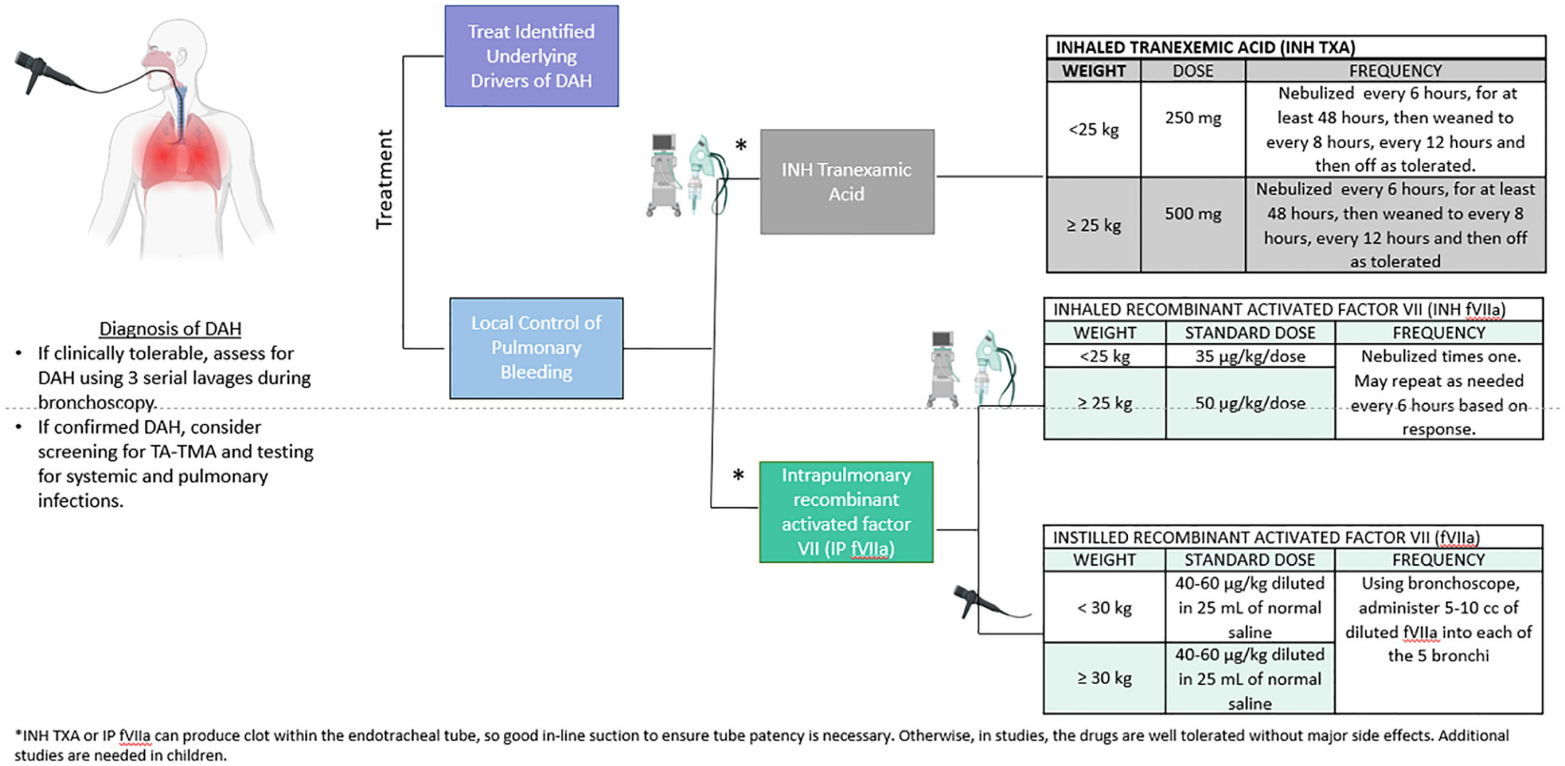
Proposed diagnosis and treatment schema with doses supported by our findings and previous literature. The ideal diagnostic approach includes bronchoscopy and evidence of persistent bleeding after 3 washes. Once DAH is confirmed, consider screening for TA-TMA, pulmonary and systemic infections and treating all identified drivers of DAH. Our data and others support treatment with INH TXA and INH or instilled recombinant active factor VIIa at the doses on the right side of the panel. While there are limited data of appropriate doses for INH TXA and intrapulmonary fVIIa for DAH, these doses were used in published in a small single center clinical trial (19) and are reported in the pediatric HCT population (17, 18, 21). In this study, only INH fVIIa was given (19), but instilled factor VIIa *via* a bronchoscope is also described (17, 18). While the data are limited, there are not severe side effects of these drugs reported in the literature. However, intrapulmonary administration of INH TXA or IP fVIIa can result in clot formation, so if patients are intubated, vigilance and intervention to ensure the tube remains patent are important.

In this multi-institutional study, INH TXA and INH fVIIa led to bleeding cessation and were associated with a decreased mortality risk. These inhaled agents can be administered *via* nebulization in most ventilators although alveolar delivery is poor with the high-frequency oscillatory ventilator (HFOV). Alternatively, these can be directly instilled in bronchi *via* bronchoscopy (**Figure 3**). Institutional preference to use HFOV could result in bias as HFOV use would preclude these therapies. While the use of HFOV was rare and similar between therapies (1/17 with steroids, 1/26 with INH TXA, 0/11 INH fVIIa), it’s possible that the severity of illness differed in other ways not captured in our data.

Children treated with steroids for any pulmonary bleed had a lower response rate and an increased risk of NRM, even after controlling for SOS, the other NRM risk factor. Our study extends the work of others that linked high-dose steroids with increased mortality in DAH post HCT (9–11).

The current paradigm of DAH pathophysiology is derived from the non-HCT setting, where alveolar damage is thought to be driven by immune-mediated mechanisms. However, 50% of children in this cohort had an identified pulmonary infection at the time of bleed, consistent with other literature in HCT (11). Further, prior studies have demonstrated that currently available diagnostic approaches to detect infections in immune compromised patients may be missing a significant number of clinically important pathogens (22). Infections can invade the endothelium directly inducing damage, and infections can worsen after corticosteroid administration. We hypothesize that infections (diagnosed and/or undiagnosed) are a key driver of the association of increased NRM and steroid treatment for DAH in the HCT setting.

Neither SOS nor defibrotide, which 18/19 (95%) of patients with SOS received, are associated with DAH. While defibrotide has a bleeding warning, in clinical trials, hemorrhagic events in patients with SOS treated with defibrotide were not significantly different than untreated patients (23). However, TA-TMA, present in 75% of our cohort, is associated with both clinical DAH and DAH on autopsy, and is increasingly being recognized as a pulmonary manifestation of TA-TMA (24–26). We noted that all autologous HCT recipients had an underlying diagnosis of neuroblastoma, and attribute this to the known association of TA-TMA and children with this disease and treatment approach (27–29). There is emerging evidence that patients with both SOS and TA-TMA are at higher risk for multi-organ failure, including DAH (25).

Given the shared endothelial injury and thrombotic changes of these three diseases, it is possible that DAH in the HCT setting may be part of a continuum of endothelial injury as most of the cohort had another concurrent endothelial syndrome, TA-TMA, SOS, or aGVHD. Endothelial damage is thought to be a major driver of other lung injuries, including COVID19 induced acute respiratory distress syndrome (30). Our data suggest that this primary endothelial injury could be a major driver in DAH post HCT which could inform treatment.

Small numbers, a retrospective approach, the lack of tissue in most patients, and the potential center effect (2 centers used INH TXA, and 1 center used INH fVIIa) are all limitations of this study. However, finding statistically significant associations with NRM in a multi- institutional study is compelling to drive future studies of treatment with INH TXA and/or INH fVIIa for DAH after HCT. While a multi-institutional large clinical trial may not be feasible, a pragmatic approach could be taken, similar to other HCT complications (31). Despite the cessation of pulmonary bleeding, outcomes remain poor in children with DAH. However, our study compares favorably to the published registry data, with 44% surviving 100 days after first bleed versus 21% (32). Our data promote local approaches to treat DAH in addition to the management of severe coincident complications, including TA-TMA, SOS, GVHD, and infections.

## Data availability statement

The raw data supporting the conclusions of this article will be made available by the authors, without undue reservation.

## Ethics statement

The studies involving human participants were reviewed and approved by IRBs at Children's Healthcare of Atlanta, University of California San Francisco, and Cincinnati Children's Medical Center. The requirement for written informed consent was waived given the low risk and the fact that most patients were deceased.

## Author contributions

MS, CCD, MZ, and KW designed the study. AH, MC, JO, CH, CCD, and MZ extracted clinical data. MS and AW completed the statistical analysis. MZ and MC designed an institutional protocol for INH fVIIa, and all authors participated in the clinical care of patients and editing of the manuscript. All authors contributed to the article and approved the submitted version.

## Supplementary material

Supplementary Figure 1 Multiple Complications of Endothelial Dysfunction 35/40 (88%) of children with DAH had another disease of endothelial dysfunction including transplant-associated thrombotic microangiopathy (TA-TMA), sinusoidal obstructive syndrome (SOS) or grade ¾ acute graft versus host disease (aGVHD). Twenty-one (60%) had multiple early endothelial diseases.
