# Supplementary figures and images for: Diffuse alveolar hemorrhage after hematopoietic cell transplantation- response to treatments and risk factors for mortality
Source: Front Oncol. 2023 Jul 20;13:1232621. doi: 10.3389/fonc.2023.1232621 (PMC10399223; doi:10.3389/fonc.2023.1232621)

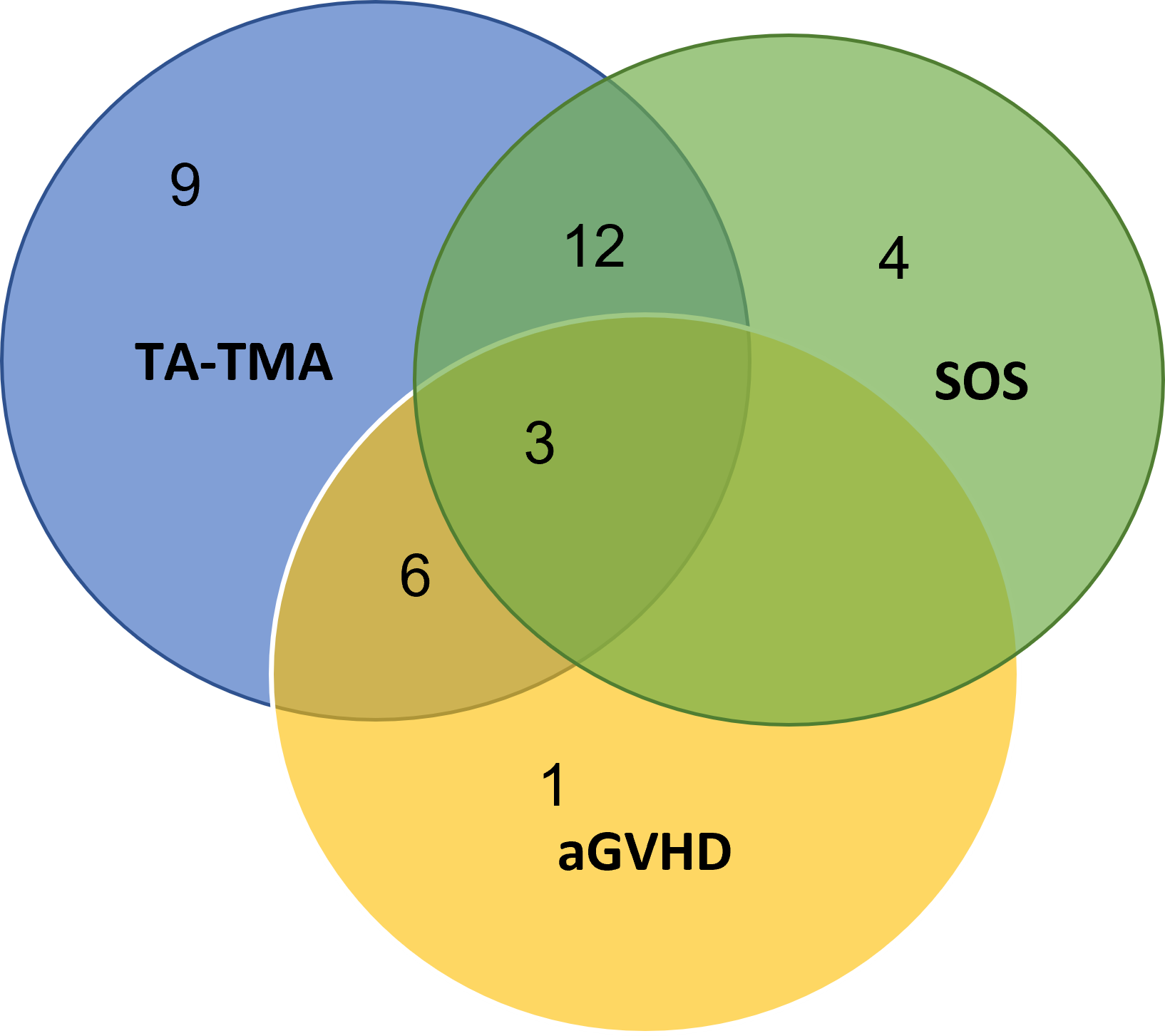

Supplement: Supplementary Figure 1 — Multiple Complications of Endothelial Dysfunction 35/40 (88%) of children with DAH had another disease of endothelial dysfunction including transplant-associated thrombotic microangiopathy (TA-TMA), sinusoidal obstructive syndrome (SOS) or grade ¾ acute graft versus host disease (aGVHD). Twenty-one (60%) had multiple early endothelial diseases. [file Image_1.tif]
